# Genetic variability in the region encompassing reiteration VII of herpes simplex virus type 1, including deletions and multiplications related to recombination between direct repeats

**DOI:** 10.1186/s40064-015-0990-y

**Published:** 2015-04-30

**Authors:** Kenichi Umene, Masami Yoshida, Yasuyuki Fukumaki

**Affiliations:** Department of Nutrition & Health Science, Faculty of Human Environmental Science, Fukuoka Woman’s University, Fukuoka, 813-8529 Japan; Department of Dermatology, Sakura Medical Center, School of Medicine, Toho University, Sakura, Chiba 285-8741 Japan; Division of Human Molecular Genetics, Center for Genetic Information, Medical Institute of Bioregulation, Kyushu University, Fukuoka, 812-8582 Japan

**Keywords:** Herpes simplex virus type 1, Nucleotide sequences, Reiteration, Direct repeats, Recombination, Deletion, Multiplication, Evolution, Molecular epidemiology

## Abstract

A number of tandemly reiterated sequences are present on the herpes simplex virus type 1 (HSV-1) DNA molecule of 152 kbp. While regions containing tandem reiterations were usually unstable, reiteration VII, which is present within the protein coding regions of gene US10 and US11, was stable; hence, reiteration VII could be used as a genetic marker. In the present study, the nucleotide sequences (159–213 bp) of a region encompassing reiteration VII of 62 HSV-1 isolates were compared with that of strain 17 as the standard strain, and the genetic variability of base substitutions, deletions, and multiplications was revealed. Base substitution was observed in nine residues on the region flanking reiteration VII and sixty-two HSV-1 isolates were classified into twelve groups based on these base substitutions. Deletions, which were present in all sixty-two isolates, were classified into six groups. Multiplications, which were present in 19 isolates having the same deletion (named del-2), were classified into four groups. The sixty-two isolates were classified into twenty patterns based on variations in the region encompassing reiteration VII, and the region encompassing reiteration VII was considered to be useful for studies on the molecular epidemiology and evolution of HSV-1. The lengths of these deletions and multiplications were multiples of 3; thus, a frame-shift mutation was not induced, and a mechanism to maintain the functions of US10 and US11 was suggested. A series of multiplications, which consisted of the duplication, triplication, and tetraplication of the same sequence, were found. Since all isolates with a multiplication had del-2, multiplications were assumed to be generated after the generation of del-2, and an isolate with del-2 was considered to have the ability to generate a multiplication. Recombination between a pair of direct repeats in and around reiteration VII was accountable for the generation of deletions and multiplications, indicating the recombinogenic property of the region encompassing reiteration VII. A correlation was revealed between a set of 20 DNA polymorphisms widely present on the HSV-1 genome and the base substitutions and deletions of the region encompassing reiteration VII, using discriminant analyses.

## Background

Herpes simplex virus (HSV), which is a widespread infectious agent in human populations and latently infects neural cells in the spinal ganglia, is classified into two serotypes, HSV-1 and HSV-2 (Nahmias et al. 2006). Lesions of HSV infections can be developed at nearly all visceral and mucocutaneous sites and the serious outcome (e.g. blindness and damage to the central nervous system) can be brought out. Genital herpes is caused by either HSV-1 or HSV-2. HSV-1 is the predominant cause of oral infection and is often acquired during childhood. The HSV-1 genome is a linear double-stranded DNA of 152 kbp, consisting of two covalently linked components, L and S (McGeoch et al. 1988; Roizman 1979). Each component consists of unique sequences flanked by inverted repeat sequences. The HSV-1 genome contains a number of different short, tandemly repeated DNA sequences (McGeoch et al. 1988; Rixon et al. 1984). Their copy numbers often vary, and variations in copy numbers can cause size heterogeneities in DNA regions containing reiterations (Maertzdorf et al. 1999; Umene 1998; Umene and Yoshida 1989).

Epidemiologically unrelated HSV-1 isolates can usually be differentiated by analyzing variations in restriction endonuclease (RE) cleavage patterns (Roizman 1979; Umene 1998). Thus, epidemiologically unrelated HSV-1 isolates can usually be differentiated by analyzing variations in RE cleavage patterns. Variations in RE cleavage patterns were divided into two types (Dean and St George 2014; Maertzdorf et al. 1999; Norberg 2010; Umene 1998; Umene and Yoshida 1989). One type, termed “restriction fragment length polymorphism (RFLP)”, was mostly due to the gain or loss of an RE cleavage site and causes a simple change in RE cleavage patterns. RFLPs are stable and serve as physical markers of the HSV-1 genome in molecular epidemiological and evolutionary studies. The other type appeared as irregularities in RE-cleaved fragments derived from certain regions of the HSV-1 genome. This variation was found in all isolates analyzed and was termed the “common type”. The common type variation was located in fragments containing tandemly repeated sequences and was due to variations in the copy number or nucleotide sequence of reiterations. The common type variation was more efficiently detected than RFLP; thus, a common type variation could be a more beneficial marker than RFLP for differentiating HSV-1 strains. However, the use of common type variations had been avoided as they may be too unstable to qualify as markers.

The degree of stability of regions containing the reiterated sequences (hypervariable regions) of the S component of the HSV-1 genome was previously examined in order to search for a common type variation that was sufficiently stable for use as a marker to distinguish HSV-1 isolates (Umene and Yoshida 1989). Reiterations I, IV, and VII were proposed to be sensitive and convenient markers for the differentiation of HSV-1 isolates. The stability of reiterations was later re-examined using polymerase chain reaction (PCR), and the usefulness of reiterations IV and VII was confirmed (Maertzdorf et al. 1999). Reiteration VII was present within the protein-coding regions of the genes US10 and US11 (McGeoch et al. 1985). The genes US10 and US11 were not essential for HSV-1 replication in cell cultures (Longnecker and Roizman 1986; Umene 1986) and were of no apparent importance for related neurovirulence or latency in mice (Nishiyama et al. 1993).

Present-day herpesviruses are regarded as descendants from a common ancestor that is supposed to have existed in the past, and are assumed to have diversified through three processes: (i) acquisition of other DNA sequences, (ii) DNA rearrangements, and (iii) base substitutions (Bowden and McGeoch 2006; Davison and McGeoch 1995; Umene and Sakaoka 1999). An HSV-1 strain is presumed to go through variations of base substitution and DNA rearrangement and to consequently diverge into distinguishable strains. Genetic variations in HSV-1 enable the differentiation and classification of isolates, thus paving the way for studies concerning (i) evolution, (ii) mode of transmission, and (iii) the relationship between isolate-specific genomic characteristics, biological characteristics, and clinical manifestations (Deback et al. 2009; Liljeqvist et al. 2009; Maertzdorf et al. 1999; Norberg 2010; Norberg et al. 2006; Roest et al. 2004; Rose and Crowley 2013; Sakaoka et al. 1994; Schmidt-Chanasit et al. 2009; Umene 1998; Umene and Kawana 2003; Umene et al. 2008; Umene et al. 2007). Variations in RE cleavage patterns between HSV-1 isolates were analyzed and a number of restriction fragment length polymorphisms (RFLPs) were detected (Norberg 2010; Norberg et al. 2006; Sakaoka et al. 1994; Umene and Yoshida 1993). HSV-1 isolates were classified into genotypes based on the condition of a set of RFLPs that were distributed widely on the whole genome of HSV-1.

In the present study, the nucleotide sequences of a region encompassing reiteration VII of a number of HSV-1 isolates were determined, and the genetic variability including deletions and multiplications besides base substitutions was revealed, indicating the usefulness of the region encompassing reiteration VII for studies on the molecular epidemiology and evolution of HSV-1. Recombination between a pair of direct repeats in and around reiteration VII was accountable for the generation of deletions and multiplications, suggesting the recombinogenic property of the region encompassing reiteration VII.

## Results and discussion

### Determination of nucleotide sequences of the region encompassing reiteration VII of HSV-1 isolates

To study the variability in the nucleotide sequences of the region encompassing reiteration VII, the DNA regions encompassing reiteration VII of fifty-eight HSV-1 isolates were amplified by PCR, and the nucleotide sequences of amplified DNAs were determined. The nucleotide sequences of the region encompassing reiteration VII of four HSV-1 isolates (Y68 (isolate 1) (Umene et al. 2007), Y70 (isolate 6) (Umene et al. 2007), C81 (Umene et al. 2009), and C85 (Umene et al. 2009)) were reported in previous studies.

### Variability in nucleotide sequences of the region encompassing reiteration VII

**Figure 1:**
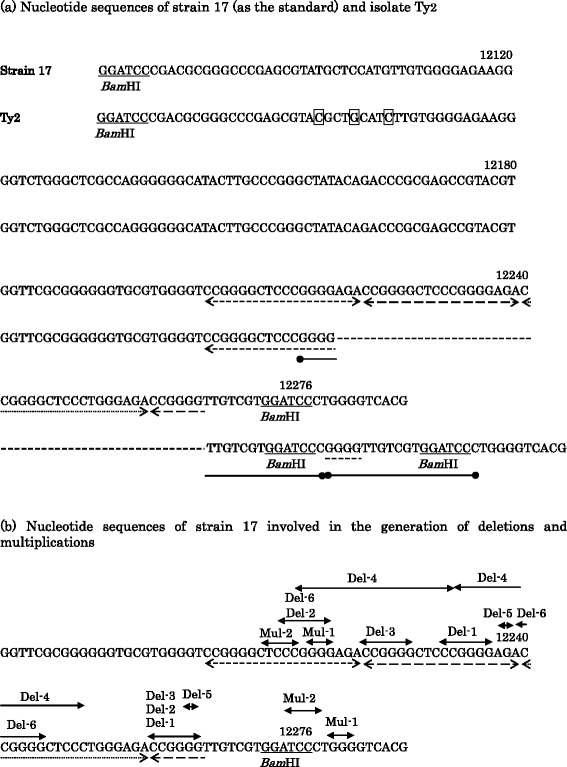
Nucleotide sequences of the region encompassing reiteration VII of HSV-1. **(a)** Nucleotide sequences of strain 17 (as the standard) and isolate Ty2. The nucleotide numbering system used is the short unique region of strain 17 (McGeoch et al. 1985). Nucleotide sequences recognized by restriction endonuclease *Bam*HI were single underlined. Each copy of the 18-bp tandem repeats of reiteration VII was underlined by a broken line having an arrowhead at both ends. Three copies of the 18-bp repeats plus 6-bp of the partial copy of reiteration VII were present in strain 17. A partial copy of 15-bp was present in reiteration VII of isolate Ty2, and no complete copy of the 18-bp repeats was present in isolate Ty2. A deletion of 45-bp (nucleotide no. 12219–12263) (del-2) found in the DNA of isolate Ty2 relative to that of strain 17 was indicated by a broken line. A duplication of 18-bp (nucleotide no. 12215–12218, and 12264–12277) (mul-2) was found in the DNA of isolate Ty2 relative to that of strain 17, and each copy of 18-bp of the duplication was indicated by a solid line having a closed circle at both ends. Each nucleotide of isolate Ty2 that differed from the corresponding nucleotide of strain 17 was enclosed within a rectangle. **(b)** Nucleotide sequences of strain 17 involved in the generation of deletions and multiplications. In the present study, six kinds of deletions (del-1 to del-6) and four kinds of multiplications (mul-1 to mul-4) were identified (Table 3). Each pair of direct repeats, which was thought to be involved in the generation of deletions and multiplications, was indicated by a pair of solid lines, each of which had an arrowhead at both ends.

The nucleotide sequences of the region encompassing reiteration VII of sixty-two isolates were compared to those of strain 17 as the standard (Table 1). The nucleotide sequence of isolate Ty2 was shown as an example (Figure 1a). Three base substitutions were found on the region flanking reiteration VII at nucleotide no. 12098, 12102, and 12106 of isolate Ty2 (Table 2). While reiteration VII of strain 17 consisted of three copies of 18-bp repeats and a partial copy of 6 nucleotides, that of isolate Ty2 was a partial copy of 15 nucleotides (Figure 1a). The complete copy of 18 bp of reiteration VII was not present on the region encompassing reiteration VII of isolate Ty2. The deletion of a 45-bp stretch (nucleotide no. 12219–12263) (del-2) was found on the DNA of the isolate Ty2 relative to that of strain 17 (Table 3). A duplication of 18-bp in length (nucleotide no. 12215–12218, and 12264–12277) (mul-2) was found on the DNA of isolate Ty2 relative to that of strain 17 (Table 3).

**Table 1:** **HSV-1 isolates analyzed**

**Isolate** ^**a**^	**Genotype** ^**b**^	**Pattern no.** ^**c**^	**Accession no. of nucleotide sequences of the region encompassing reiteration VII**
K47	F1	1	AB856424
K94	F2	1	AB856425
K41	F4	1	AB856426
Ty179	F6	1	AB856427
K90	F11	1	AB856428
Ty127	F11	1	AB856429
Ty170	F21	1	AB856430
K44	F22	1	AB856431
Ty157	F23	1	AB856432
K40	F24	1	AB856433
Ty24	F26	1	AB856434
K46	F34	1	AB856435
K59	F11	2	AB856436
C85	F85	3	AB426486
K79	F1	4	AB856437
K93	F1	4	AB856438
K82	F1	4	AB856439
Ty145	F1	4	AB856440
Ty148	F1	4	AB856441
Ty16	F13	4	AB856442
K26	F27	4	AB856443
Ty14	F30	4	AB856444
Ty35	F30	4	AB856445
Ty128	F33	4	AB856446
Ty2	F1	5	AB856447
Ty15	F1	5	AB856448
Ty23	F7	5	AB856449
Ty3	F9	5	AB856450
K84	F17	5	AB856451
K80	F26	5	AB856452
Ty21	F29	5	AB856453
K60	F31	5	AB856454
Ty1	F31	5	AB856455
Ty109	F25	6	AB856456
Ty102	F14	7	AB856457
Ty119	F16	7	AB856458
K76	F16	8	AB856459
Ty153	F16	9	AB856460
K57	F1	10	AB856461
Ty43	F4	10	AB856462
K54	F5	10	AB856463
K63	F15	10	AB856464
Ty149	F25	10	AB856465
Ty169	F28	10	AB856466
K53	F32	10	AB856467
Ty44	F35	10	AB856468
K86	F35	10	AB856469
C81	F35	10	AB426482
K81	F1	11	AB856470
K78	F35	12	AB856471
Ty161	F17	13	AB856472
Ty25	F35	13	AB856473
Ty106	F35	13	AB856474
Ty129	F35	13	AB856475
K43	F8	14	AB856476
K42	F19	15	AB856477
K56	F20	16	AB856478
Ty141	F25	17	AB856479
Ty98	F12	18	AB856480
Y68 (isolate 1)	F83	18	AB266255
K8	F35	19	AB856481
Y70 (isolate 6)	F84	20	AB266260

^a^The nucleotide sequences of the region encompassing reiteration VII of 62 HSV-1 isolates listed in Table 1 were examined in the present study.

^b^The genotypes of 62 HSV-1 isolates listed in Table 1 were previously determined on the basis of the situation of 20 RFLPs, which were distributed on the L component of HSV-1 DNA (Umene et al. 2009; Umene et al. 2007; Umene and Yoshida 1993).

^c^The 62 HSV-1 isolates listed in Table 1 were classified into twenty groups, named pattern no. 1 (PN-1) to 20 (PN-20), on the basis of the nucleotide sequences of the region encompassing reiteration VII (Table 2).

**Table 2:** **Pattern no. of nucleotide sequences of the region encompassing reiteration VII**

**Pattern no.** ^**a**^ **(No. of isolates)**	**Nucleotide no. of base substitutions** ^**b**^											**Deletion** ^**c**^	**Multiplication** ^**d**^
	**12082**	**12098**	**12102**	**12106**	**12110**	**12173**	**12174**	**12185**	**12193**	**12221**	**12234**		
**Strain 17 as the standard**	**C**	**T**	**C**	**G**	**T**	**C**	**C**	**C**	**G**	**A**	**G**		
1 (12)	+	+	+	+	+	+	+	+	+	+	+	4	
2 (1)	+	+	+	+	+	+	+	+	+	+	np	3	
3 (1)	+	+	+	C	+	+	+	+	+	np	np	6	
4 (10)	+	C	G	C	+	+	+	+	+	G	+	1	
5 (9)	+	C	G	C	+	+	+	+	+	np	np	2	2
6 (1)	+	C	G	C	+	+	+	+	+	np	np	2	3
7 (2)	+	+	G	C	G	+	+	+	+	np	np	2	
8 (1)	+	+	G	C	G	+	+	+	+	np	np	2	2
9 (1)	+	+	G	C	G	+	+	+	+	np	np	2	4
10 (10)	+	C	G	C	+	+	+	T	+	G	+	1	
11 (1)	+	C	G	C	+	+	+	T	+	np	np	2	1
12 (1)	+	C	G	C	+	+	+	T	+	np	np	2	
13 (4)	+	C	G	C	G	+	+	+	+	np	np	2	2
14 (1)	+	C	G	C	+	T	+	+	+	G	A	1	
15 (1)	+	C	G	C	+	T	+	+	+	G	+	5	
16 (1)	+	C	G	C	+	+	T	+	+	np	np	2	2
17 (1)	T	C	G	C	+	+	+	+	+	np	np	2	2
18 (2)	+	C	G	C	+	+	+	T	C	G	+	1	
19 (1)	T	C	G	C	+	+	+	T	+	G	+	1	
20 (1)	+	C	G	C	G	+	+	T	+	np	np	2	

^a^The nucleotide sequences of the region encompassing reiteration VII were classified into twenty groups, named pattern no. 1 (PN-1) to 20 (PN-20), and indicated as 1 to 20, respectively. The nucleotide sequences of strain 17 were used as the standard (McGeoch et al. 1985). “+” indicates “the same as that of strain 17”. “np” indicates “not present” due to deletions.

^b^The nucleotide numbering system used was a short unique region of HSV-1 strain 17 (McGeoch et al. 1985).

^c^Six kinds of deletions were found, named del-1 to del-6 (Table 3), and indicated as 1 to 6, respectively.

^d^Four kinds of multiplications were found, named mul-1 to mul-4 (Table 3), and indicated as 1 to 4, respectively.

**Table 3:** **Deletions and multiplications**

**Name** ^**a**^	**Definition** ^**b**^	**Pair of direct repeats** ^**c**^	**No. of isolates**
Del-1	Deletion of 27 bp (12237–12263), from the 16th residue of the 2nd element of reiteration VII to the 6th (the last) residue of the 4th element	CCGGGG	24
		12231–12236	
		12258-12263	
Del-2	Deletion of 45 bp (12219–12263), from the 16th residue of the 1st element of reiteration VII to the 6th (the last) residue of the 4th element	CCGGGG	23
		12213–12218	
		12258-12263	
Del-3	Deletion of 36 bp (12228–12263), from the 7th residue of the 2nd element of reiteration VII to the 6th (the last) residue of the 4th element	CCGGGG	1
		12222–12227	
		12258-12263	
Del-4	Deletion of 18 bp (12233–12250), from the 12th residue of the 2nd element of reiteration VII to the 11th residue of the 3rd element	GGGGAGACCGGGGCTCCC	12
		12215–12232	
		12233-12250	
Del-5	Deletion of 24 bp (12239–12262), from the 18th (the last) residue of the 2nd element of reiteration VII to the 5th residue of the 4th element	G	1
		12238	
		12262	
Del-6	Deletion of 27 bp (12219–12245), from the 16th residue of the 1st element of reiteration VII to the 6th residue of the 3rd element	CCGGGG	1
		12213–12218	
		12240-12245	
Mul-1	Duplication of 18 bp (12264–12281), from the 1st residue of the region surrounding the 4th element of reiteration VII	GGG	1
		12216–12218	
		12279-112281	
Mul-2	Duplication of 18 bp (12215–12218, 12264–12277), from the 12th residue of the 1st element of reiteration VII	TCCC	16
		12211–12214	
		12274-12277	
Mul-3	Triplication of 18 bp (12215–12218, 12264–12277), from the 12th residue of the 1st element of reiteration VII	TCCC	1
		12211–12214	
		12274-12277	
Mul-4	Tetraplication of 18 bp (12215–12218, 12264–12277), from the 12th residue of the 1st element of reiteration VII	TCCC	1
		12211–12214	
		12274-12277	

^a^Six kinds of deletions were found on the nucleotide sequences encompassing reiteration VII relative to those of strain 17, and were named del-1 to del-6. Four kinds of multiplications were found on the nucleotide sequences encompassing reiteration VII relative to those of strain 17, and named mul-1 to mul-4.

^b^The nucleotide numbering system used was a short unique region of HSV-1 strain 17 (McGeoch et al. 1985).

^c^The nucleotide sequences of each pair of direct repeats, which were assumed to be involved in the generation of deletions or multiplications, and the nucleotide no. of each pair were indicated.

Base substitution was observed in nine residues on the region flanking reiteration VII (Table 2). Sixty-two HSV-1 isolates were classified into twelve groups based on these base substitutions. All sixty-two HSV-1 isolates had a deletion. Six kinds of deletions were detected and named del-1 to del-6 (Table 3). The lengths of these deletions were 18, 24, 27, 36, and 45 bp, which were multiples of 3; thus, a frame-shift mutation was not induced. Nineteen isolates had a multiplication (namely, duplication, triplication, and tetraplication). All 19 isolates with a multiplication had del-2. Four kinds of multiplications were detected and named mul-1 to mul-4 (Table 3). The lengths of these multiplications were 18, 36, and 54 bp, which are multiples of 3; thus, a frame-shift mutation was not induced. The same stretch was multiplied twice (mul-2), three times (mul-3), and four times (mul-4).

Methods to discriminate HSV-1 isolates can be used to address questions regarding recrudescent lesions (which are caused by endogenous recurrence or exogenous re-infection) and modes of transmission (e.g. in cases of nosocomial infections). The region of HSV-1 DNA used to discriminate HSV-1 isolates should show a considerable degree of variability. The regions encompassing reiteration VII of the 62 HSV-1 isolates examined in the present study were classified into twenty groups, named pattern no. 1 (PN-1) to 20 (PN-20), on the basis of the states of base substitution, deletion, and multiplication (Table 2). A large degree of variability was revealed on the region encompassing reiteration VII; thus, determining the nucleotide sequences of the region encompassing reiteration VII can be useful for discriminating HSV-1 isolates. HSV-1 isolates having the same nucleotide sequences of the region encompassing reiteration VII often differed in RELPs (Table 1).

A set of four HSV-1 isolates of C81, C82, C83, and C84 were obtained sequentially from one patient, and the other set of four HSV-1 isolates of C85, C86, C87, and C88 were obtained sequentially from the other patient in a previous study (Umene et al. 2009). While RFLPs of HSV-1 isolates obtained from one patient were different from those from the other patient, RFLPs of HSV-1 isolates obtained sequentially from the same patient were the same. The recrudescent lesions of the two patients were considered to be attributed to endogenous recurrence of a latent virus. The nucleotide sequences of the region encompassing reiteration VII of four HSV-1 isolates of C81, C82, C83, and C84 were the same, and the nucleotide sequences of the region encompassing reiteration VII of four HSV-1 isolates of C85, C86, C87, and C88 were the same. Thus, when the RFLPs of HSV-1 isolates separated from the same patient were the same, the nucleotide sequences of the region encompassing reiteration VII of these isolates were assumed to be usually the same. The nucleotide sequences of the region encompassing reiteration VII seemed to be useful for studies on molecular epidemiology and evolution of HSV-1; however, a number of other genetic markers were assumed to be required for the differentiation and classification of HSV-1 strains in addition to the reiteration VII region.

The degree of stability of regions containing the reiterated sequences (hypervariable regions) of the S component of the HSV-1 genome was previously examined, and reiterations IV and VII were proposed to be sensitive and convenient markers for the differentiation of HSV-1 isolates (Maertzdorf et al. 1999; Umene and Yoshida 1989). The variability in the length of reiteration IV region appeared to be higher than that in the reiteration VII region (Maertzdorf et al. 1999); thus, a molecular analysis method involving amplification of reiteration IV region was developed (Dean and St George 2014). Reiteration IV is located within introns of both the genes US1 and US12 on the inverted repeat sequences; hence, reiteration IV is present twice within the HSV-1 genome. Recombination between inverted repeat sequences including introns was assumed to occur, probably causing variations. In a previous study, differences in length of reiteration IV region were detected between HSV-1 isolates from the same patient: however, differences in length of reiteration VII region were not evident; hence, reiteration VII appeared to be more stable than reiteration IV (Umene and Kawana 2003). In the present study, nucleotide sequences of the region encompassing reiteration VII were determined and studied because of the stability of reiteration VII.

The presence of hypervariable regions in the HSV-2 genome has not been definitively established (Martin et al. 2006). Microsatellites, which are short tandem repeats of 1 to 6 bp and are highly variable and polymorphic, were present on the HSV-1 and HSV-2 genomes (Burrel et al. 2013; Deback et al. 2009). They were reported to be used as markers for the differentiation of HSV-1 and HSV-2 strains. In comparison with reiterated regions in the S component, microsatellites have the advantage of being distributed across all parts of the HSV genome. Changes in repeat number at microsatellite loci and on reiteration VII is a mechanism of variability of microsatellites and reiteration VII. The detection of changes in repeat number on reiteration VII was assumed to be easier than that of microsatellite loci, because the length of element of reiteration VII (18 bp) is longer than that of microsatellites (1 to 6 bp).

### Generation of deletions and multiplications

A number of deletions and multiplications were identified; however, no frame-shift mutation was present. Maertzdorf et al. suggested that reiteration VII was a target for selective pressure since it was located within a protein-coding region (Maertzdorf et al. 1999). Thus, a mechanism to maintain the functions of US10 and US11 was presumed to have occurred during HSV-1 infections in humans, although the genes of US10 and US11 were not essential for HSV-1 replication in cell cultures (Longnecker and Roizman 1986; Umene 1986) and were of no apparent importance for related neurovirulence or latency in mice (Nishiyama et al. 1993). Molecular epidemiological studies may be able to reveal findings that may not be clearly revealed by model systems, such as cell culture and experimental animal studies.

Gene duplication is an important process supporting the functional diversification of genes in the evolutionary change of DNA (Liberles et al. 2010; Nei 1987). Genes with improved function may arise by the elongation of genes, and gene elongation is usually caused by the duplication of a gene or part of a gene. Reiteration VII of strain 17 consisted of three copies of the 18-bp sequence plus a partial copy of 6-bp (McGeoch et al. 1985; Rixon et al. 1984). Del-2 was the longest of the 6 kinds of deletions (Table 3). Reiteration VII of the isolate with del-2 was 15-bp in length and did not have a complete copy of reiteration VII; hence, the complete copy of reiteration VII was considered to be non-essential for HSV-1 growth. Since all isolates with a multiplication had del-2 (Table 2), multiplications were assumed to be generated after the generation of del-2, and an isolate with del-2 was considered to have the ability to generate a multiplication, which elongated the region of reiteration VII to compensate for shortening of the region of reiteration VII due to del-2 (Nei 1987).

**Figure 2:**
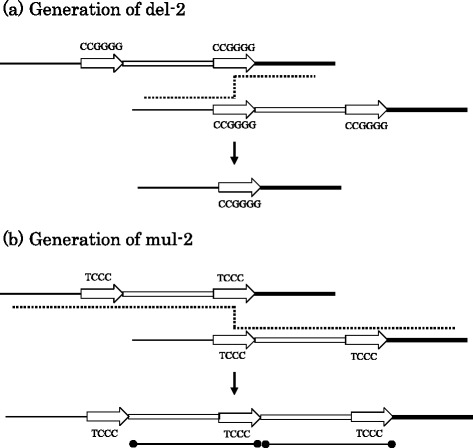
Involvement of a pair of direct repeats in the generation of deletions or multiplications. Direct repeats on the region encompassing reiteration VII were indicated by horizontal arrows, and the stretch flanked by a pair of direct repeats was indicated by a double line. **(a)** Generation of del-2. The misalignment of direct repeats (CCGGGG) during DNA replication indicated by a broken line could result in the generation of the deletion of del-2. **(b)** Generation of mul-2. The misalignment of direct repeats (TCCC) during DNA replication indicated by a broken line could result in the generation of the multiplication of mul-2 (duplication). Each copy of the duplicated sequences was indicated by a solid line having a closed circle at both ends.

Direct repeats are identical or closely related DNA sequences present in two or more copies in the same orientation in the same molecule, although not necessarily adjacent (King et al. 2006). Recombination between direct repeats on misaligned molecules can generate genetic diversity, and the involvement of direct repeats with the generation of deletions and multiplications on the region encompassing reiteration VII was suggested (Emanuel and Shaikh 2001; Treangen et al. 2009). The nucleotide sequence of the region encompassing reiteration VII of isolate Ty2 having a deletion of del-2 and a multiplication of mul-2 was shown as an example (Figure 1a). A 6-bp sequence, “CCGGGG” (nucleotide no. 12213–12218), adjoined the left end of the deleted 45-bp sequence of del-2, and the same sequence, “CCGGGG” (nucleotide no. 12258–12263), was present on the right end of the deleted 45-bp sequence; hence, a pair of direct repeats of “CCGGGG” were present in relation to del-2. The generation of del-2 was attributable to recombination between the pair of direct repeats of “CCGGGG” (Figure 2a). Similar to the case of del-2, a pair of direct repeats was found in cases of other deletions (Figure 1b, Table 3). The sequence “CCGGGG” on the reiteration VII appeared to be involved in the generation of the deletions del-1, del-2, del-3, and del-6, suggesting the recombinogenic property of the sequence “CCGGGG”.

A 4-bp sequence, “TCCC” (nucleotide no. 12211–12214), adjoined the left end of the duplicated 18-bp sequence of mul-2, and the same sequence, “TCCC” (nucleotide no. 12274–12277), was present on the right end of the duplicated 18-bp sequence; hence, a pair of direct repeats of “TCCC” were present in relation to mul-2 (Figure 1a, b). The generation of mul-2 was attributable to recombination between the pair of direct repeats of “TCCC” (Figure 2b). Mul-3 and mul-4 were a triplication and tetraplication of the 18-bp sequence similar to the duplicated sequence of mul-2, respectively. The generation of mul-3 and mul-4 was attributable to recombination between the pair of direct repeats of “TCCC”, similar to mul-2. Another pair of direct repeats of the 3-bp sequence “GGG” were present in relation to the duplication of mul-1. Thus, recombination between a pair of direct repeats was assumed to be involved in the generation of multiplications in a manner similar to the generation of deletions, on the region encompassing reiteration VII of HSV-1, indicating the importance of direct repeats in DNA rearrangement.

### Relationship between the set of 20 RFLPs and nucleotide sequences of the region encompassing reiteration VII

The relationship between the set of 20 RFLPs and nucleotide sequences of the region encompassing reiteration VII was examined. A discriminant analysis can be used to determine the group (e.g. “pattern no.”) to which an individual (e.g. 62 HSV-1 isolates) belongs based on the characteristics of that individual (e.g. the set of 20 RFLPs) (Huberty 1994; Sharma 1996). The classification results obtained by the discriminant analysis show how well group membership (e.g. “pattern no.”) (coming under the criterion variable) can be predicted using the set of 20 RFLPs (coming under predictor variables). The classification results by prediction may include cases misclassified in addition to cases correctly classified.

The 62 isolates examined in the present study (the real sample) were classified into twenty groups of PN-1 to PN-20 (“pattern no.”, corresponding to the criterion variable) (Table 2). Predictor variables were the set of 20 RFLPs, which were distributed widely on the L component of HSV-1 DNA (Umene and Yoshida 1993). A discriminant analysis was conducted to the real sample using JMP10 statistical discovery software (Lehman et al. 2005; Sall et al. 2005). The number of cases correctly classified was 36, and that misclassified was 26 (=62-36).

It remained unknown whether a correlation existed between the set of 20 RFLPs and “pattern no.” in the real sample. A hypothetical random sample consisting of 62 hypothetical isolates, each of which had two characteristics of the genotype (corresponding to each set of 20 RFLPs) and “pattern no.”, was generated by randomly selecting the genotype and “pattern no.” from the real sample sixty-two times (Efron and Tibshirani 1998; Mooney and Duval 1993). The “pattern no.” of each hypothetical isolate of this hypothetical random sample was selected independently of the genotype; thus, a correlation between the genotype and “pattern no.” was assumed to be absent and the accuracy of the classification was considered to generally be low. Discriminant analyses were conducted to the fifty hypothetical random samples. The 95th “percentile point in the distribution of the numbers of cases correctly classified of fifty discriminant analyses of hypothetical samples” (abbreviated to “PDNCH”) was calculated. If a correlation was present between predictor variables and the criterion variable in the real sample, “the number of cases correctly classified of discriminant analysis of the real sample” (abbreviated to “NCR”) was assumed to be more than the 95th PDNCH (Kazmier 1996; Spiegel and Stephens 1998). In discriminant analyses using “pattern no.” as the criterion variable, NCR was 36, which was less than 38, the 95th PDNCH. Therefore, a correlation between the set of 20 RFLPs and “pattern no.” was considered to be absent in the real sample.

Discriminant analyses of each of “base substitution”, “deletion”, and “multiplication” as the criterion variable were conducted using the genotype as predictor variables. In the discriminant analysis of the real sample using “base substitution” as the criterion variable, NCR was 44, which was more than 41, the 95th PDNCH; hence, a correlation appeared to be present between the set of 20 RFLPs and “base substitution” in the real sample. The set of base substitutions, which were detected as RFLPs on the L component, were considered to be associated with another set of base substitutions on the region encompassing reiteration VII on the S component. In the discriminant analysis of the real sample using “deletion” as the criterion variable, NCR was 44, which was more than 43.5, the 95th PDNCH; hence, a correlation appeared to be present between the set of 20 RFLPs and “deletion” in the real sample. Each deletion on the region encompassing reiteration VII was assumed to have been generated in an ancestor virus with a set of RFLPs, and the deletions generated were assumed to be transmitted to progeny, as well as the set of RFLPs, thereby maintaining a correlation between the set of 20 RFLPs and “deletion” on the region encompassing reiteration VII. “Base substitution” and “deletion” of reiteration VII region were assumed to be useful as a genetic marker correlated with the set of RFLPs.

In the discriminant analysis of the real sample using “multiplication” as the criterion variable, NCR was 38, which was less than 52.5, the 95th PDNCH; hence, a correlation did not appear to be present between the set of 20 RFLPs and “multiplication” in the real sample. Norberg et al. classified HSV-1 isolates into three groups, based on the nucleotide sequences of genes encoding glycoproteins: no relationship was found between the number of repeated blocks of gene encoding glycoprotein I and classification into the phylogenetically separated groups, suggesting that the tandem repeat region evolved separately from and faster than the remaining part of the gene (Norberg et al. 2004). Although a correlation did not appear to be present between the set of 20 RFLPs and “pattern number” and between the set of 20 RFLPs and “multiplication” in the real sample, “pattern number” and “multiplication” of reiteration VII region were assumed to be useful as a genetic marker for differentiation of HSV-1 isolates.

Genetic recombination is the occurrence of progeny with combinations of genes other than those that occurred in the parents due to independent assortment or crossing over (King et al. 2006; Leach 1996; Umene 1999). The occurrence of recombination between the viruses of different genetic groups was suggested in sequencing studies of different regions on the genomes of a large number of HSV-1isolates (Bowden et al. 2004; Norberg et al. 2004). Kolb et al. found that the majority of recombination that was detected across the entirety of the tree occurred near the root nodes (Kolb et al. 2013). Once the individual strains began diverging, there was little evidence for recombination, suggesting that recombination did not significantly disrupt the clade structure and is not a confounding factor (Kolb et al. 2013). HSV-1 is thought to have co-migrated and diversified with its human host (Bowden et al. 2006; Bowden and McGeoch 2006; Kolb et al. 2013; Sakaoka et al. 1994; Umene and Sakaoka 1997, 1999). Szpara et al. found that the HSV-1 strains analyzed clustered by geographic origin during whole-genome distance analysis, while recombination occurred with high frequency throughout the HSV-1 genome (Szpara et al. 2014). In the present study, the association between the set of 20 RFLPs on the L component and “base substitution” and “deletion” on the region encompassing reiteration VII on the S component appeared to remain, while the degree of this association may have been decreased by the occurrence of recombination. Although the region encompassing reiteration VII was presumed to be useful as a genetic marker for HSV-1, the use of a number of other genetic markers distributed widely on the HSV-1 genome was assumed to be required for the differentiation and classification of HSV-1 isolates.

## Conclusions

A large degree of variability including deletions and multiplication was revealed on a region encompassing reiteration VII of HSV-1 by determination of the nucleotide sequences of the region encompassing reiteration VII of a number of HSV-1 isolates. Recombination between a pair of direct repeats in and around reiteration VII was assumed to be involved in the generation of deletions and multiplications, suggesting the recombinogenic property of the region encompassing reiteration VII. The region encompassing reiteration VII was considered to be useful for studies on the molecular epidemiology and evolution of HSV-1.

## Methods

### Viruses

The nucleotide sequences of a region encompassing reiteration VII of 62 HSV-1 isolates were examined in the present study (Table 1), and these HSV-1 isolates were classified into genotypes based on the state of a set of 20 RFLPs in previous studies (Umene and Yoshida 1993). Fifty-eight HSV-1 isolates, prefixed with “Ty” or “K”, were epidemiologically unrelated, and the nucleotide sequences of the region encompassing reiteration VII of these fifty-eight isolates were determined in the present study (Umene and Yoshida 1993). The nucleotide sequences of the region encompassing reiteration VII of four HSV-1 isolates (Y68 (isolate 1) (Umene et al. 2007), Y70 (isolate 6) (Umene et al. 2007), C81 (Umene et al. 2009), and C85 (Umene et al. 2009)) were reported in previous studies. C81 and C85 were isolated from different individuals (Umene et al. 2009). Y68 (isolate 1) and Y70 (isolate 6) were isolated from the same individual; however, Y68 (isolate 1) and Y70 (isolate 6) differed in RFLPs (Umene et al. 2007). The case of Y68 (isolate 1) and Y70 (isolate 6) was assumed to be due to exogenous re-infection (Umene et al. 2007).

Working stocks of HSV-1 were made on Vero cells in Eagle’s MEM with 2% fetal bovine serum at a low multiplicity of infection. HSV-1 DNA was prepared from viral particles as described (Umene and Yoshida 1989).

### Polymerase chain reaction (PCR) and sequencing

PCR to amplify the region encompassing reiteration VII was performed using a pair of primers: 5′-GTGGGTTGGGCTTCCGGTGG-3′ (nucleotide number 12032–12051) and 5′-CCAGAGACCCCAGGGTACCG-3′ (12288–12307). The nucleotide numbering system was a short unique region of HSV-1 strain 17 (McGeoch et al. 1985). DNA polymerase for PCR (*PrimeSTAR GXL DNA Polymerase*) was purchased from Takara Bio Inc. (Otsu, Japan). The amplification program started with an initial denaturation of 30 seconds at 98°C, followed by 30 cycles of amplification consisting of 10 seconds of denaturation at 98°C, and 20 seconds of annealing and elongation at 72°C. The PCR product by this method was detected as one band in acrylamide gel electrophoresis. The sequencing of DNA products by PCR was carried out on an automated sequencer.

### Statistical analysis

The data files analyzed in this study were created and edited using Microsoft Access 2010 on the basis of the relational database model (Mata-Toledo and Cushman.P.K. 2000; Smith and Sussman 2003). The statistical software JMP10 (SAS Institute Inc.) was used for statistical analyses (Kazmier 1996; Lehman et al. 2005; Sall et al. 2005; Spiegel and Stephens 1998).

### Accession numbers of nucleotide sequences

The nucleotide sequences of the region encompassing reiteration VII of fifty-eight HSV-1 isolates, corresponding to the short unique region between 12073 and 12276 of strain 17 (between 12073 and 12281 in the case of isolate K81), were determined in the present study and were submitted to DDBJ/EMBL/GenBank. The accession numbers were AB856424 to AB856481 (Table 1). Those of Y68 (isolate 1) (Umene et al. 2007), Y70 (isolate 6) (Umene et al. 2007), C81 (Umene et al. 2009), and C85 (Umene et al. 2009) were reported in previous studies (Table 1).

## Abbreviations

HSV: Herpes simplex virus
HSV-1: Herpes simplex virus type 1
HSV-2: Herpes simplex virus type 2
RE: Restriction endonuclease
RFLP: Restriction fragment length polymorphism
PCR: Polymerase chain reaction
PDNCH: Percentile point in the distribution of the numbers of cases correctly classified of fifty discriminant analyses of hypothetical samples
NCR: The number of cases correctly classified of discriminant analysis of the real sample
